# A Unique Nest-Protection Strategy in a New Species of Spider Wasp

**DOI:** 10.1371/journal.pone.0101592

**Published:** 2014-07-02

**Authors:** Michael Staab, Michael Ohl, Chao-Dong Zhu, Alexandra-Maria Klein

**Affiliations:** 1 Department of Nature Conservation and Landscape Ecology, Institute of Earth and Environmental Sciences, University of Freiburg, Freiburg, Germany; 2 Ecosystem Function Group, Institute of Ecology, University of Lüneburg, Lüneburg, Germany; 3 Museum für Naturkunde, Leibniz Institute for Research on Evolution and Biodiversity, Berlin, Germany; 4 Key Laboratory of Zoological Systematics and Evolution, Institute of Zoology, Chinese Academy of Sciences, Beijing, P.R. China; Universidade de São Paulo, Faculdade de Filosofia Ciências e Letras de Ribeirão Preto, Brazil

## Abstract

Hymenoptera show a great variation in reproductive potential and nesting behavior, from thousands of eggs in sawflies to just a dozen in nest-provisioning wasps. Reduction in reproductive potential in evolutionary derived Hymenoptera is often facilitated by advanced behavioral mechanisms and nesting strategies. Here we describe a surprising nesting behavior that was previously unknown in the entire animal kingdom: the use of a vestibular cell filled with dead ants in a new spider wasp (Hymenoptera: Pompilidae) species collected with trap nests in South-East China. We scientifically describe the ‘Bone-house Wasp’ as *Deuteragenia ossarium* sp. nov., named after graveyard bone-houses or ossuaries. We show that *D. ossarium* nests are less vulnerable to natural enemies than nests of other sympatric trap-nesting wasps, suggesting an effective nest protection strategy, most likely by utilizing chemical cues emanating from the dead ants.

## Introduction

Natural selection of life-history strategies results in increased individual fitness by ensuring successful reproduction, but reproductive strategies in animals vary widely [1], [2]. While r-strategists produce a high number of offspring with limited survival chances, offspring of K-strategists have much higher survival chances at the cost of limited reproductive potential. Hymenoptera show a broad variation in reproductive potential, making them ideal models to study reproductive strategies [3]. Typical for most insects, a single female of sawflies and parasitic wasps, which represent the earliest branches in the phylogeny, can lay hundreds of eggs during her lifetime, but the more advanced solitary nest-provisioning Hymenoptera can rarely lay more than a dozen (reviewed in O’Neill [3]) with some species having even lower reproductive rates [4].

Examples of the latter group are the Pompilidae, a cosmopolitan family of spider-hunting wasps, of which the most well-known members are the eye-catching tarantula hawks of the New World genus *Pepsis* [5]. Currently about 5000 predominately tropical and subtropical species are described [6]. The larvae of almost all species develop on a single paralyzed spider while the adults feed mostly on floral nectar [3]. Pompilidae show a wide range of reproductive strategies from ectoparasitoids and cleptoparasitoids to nest-provisioning predators [3], [7]. Most species are solitary nesters but parasocial examples are known [8], [9]. In the nest-provisioning Pompilidae the nesting type is a variable albeit crucial aspect of the life history as the nest is primarily protecting the offspring against natural enemies (predators and parasitoids). Usually nests are excavated in the soil, but some species occupy pre-existing above-ground cavities such as abandoned galleries of wood-dwelling beetles [3], [10], [11]. Mud cells attached under leafs or rock prominences as well as free-hanging nests coated with plant resin occur in several species [7], [12].

Species of the genus *Deuteragenia* Šustera, 1912 in the subfamily Pepsinae show the usual nest-construction behavior of cavity-nesting Pompilidae. As far as it is known, nests contain several brood cells separated by thin walls of plant debris, resin or, most commonly, soil material out of which the empty outermost vestibular cell is constructed once food-provisioning and egg-laying are completed [3], [10], [11]. After nest construction, nests are abandoned and the females do not further care for the offspring. Hence, there is no protection for the developing larvae against natural enemies except the fragile physical barrier of the vestibular cell. *Deuteragenia* comprises more than 50 species and has a worldwide distribution in wooded areas with the exception of Australia [11], [13], [14]. The *Deuteragenia* fauna occurring in Japan and the Russian Far East has been revised in detail [11], [15], [16].

In this paper we describe *Deuteragenia ossarium* sp. nov. from South-East China. The species is unique not only in the genus *Deuteragenia* but also in Pompilidae and all other cavity-nesting wasps by showing a novel and unique nest-protection strategy, the construction of a vestibular cell filled with dead ants.

## Materials and Methods

### Ethics Statement

Necessary permits to conduct field work were obtained from the Administration Bureau of the Gutianshan National Nature Reserve.

### Study site

This study was carried out in the Gutianshan National Nature Reserve (GNNR) (29°14′ N/118°07′ E), Zhejiang Province, South-East China [17], [18]. The GNNR was established in 1975 to protect about 8,000 ha of a highly diverse semi-evergreen broad-leaved forest. The terrain is characterized by steep slopes along an elevation gradient between 250 and 1260 m above sea level. The mean annual temperature is 15.3°C and the mean annual precipitation is 1964 mm [19]. Typical tree species in old-growth forest are *Castanopsis eyrei* (Champion ex Benth.) Hutch. (Fagaceae), *Cyclobalanopsis glauca* (Thunberg) Oers. (Fagaceae), and *Schima superba* Gardn. et Champion (Thecaceae) [17], [18]. Outside the GNNR, monocultures of the conifers *Cunninghamia lanceolata* (Lamb.) Hook. (Cupressaceae) and *Pinus massoniana* (Lamb.) (Pinaceae) dominate [20].

In 2008, the ‘BEF-China’ project established a total of 27 study plots across the entire GNNR, each comprising 30 m×30 m, along gradients of tree species richness and forest age that we worked on in this study. For detailed descriptions of the study plots including maps, geological information and exhaustive botanical details we refer to Bruelheide et al. [17].

### Sample and data collection

**Figure 1:**
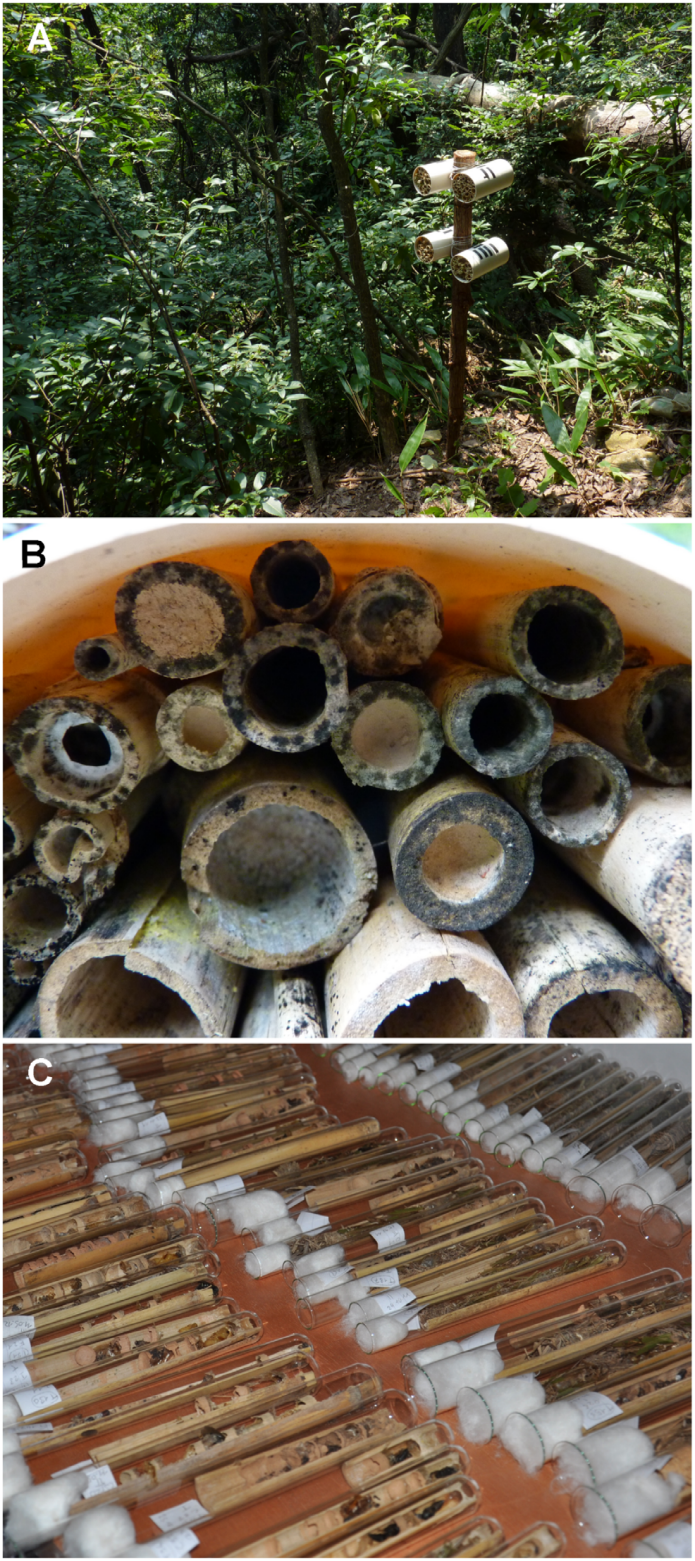
Trap nests to collect solitary cavity-nesting Hymenoptera. (A) Exposed trap nest in the Gutianshan National Nature Reserve, the type locality of *Deuteragenia ossarium*. (B) Occupied reed internodes containing nests are identifiable by the characteristic nest seal that, in most species, consists of soil material. (C) Opened nests reared in test tubes closed with cotton wool. Photographs: Michael Staab.

Nests of solitary cavity-nesting wasps were collected in the 27 plots with trap nests from September 2011 to October 2012. In each plot, two wooden posts with four trap nests, each consisting of a plastic tube (length: 22 cm, diameter: 12.5 cm) filled with *Arundo donax* L. (Poaceae) internodes (diameter: 2–20 mm) were exposed (Figure 1A). Trap nests were placed at approximately 1.5 m height to provide standardized nesting sites for cavity-nesting solitary wasps [21], [22]. Monthly, internodes containing nests that are easily distinguishable by the characteristic nest-closing plug (Figure 1B) were collected and a fungicide that is non-toxic to Hymenoptera (Folicur, Bayer CropScience, Monheim, Germany) was applied with a hand sprayer to prevent molding in the humid climate of the GNNR. Nests were taken to the laboratory, opened, and reared in glass test tubes closed with cotton wool (Figure 1C) until eclosion.

For every nest we recorded the number of brood cells and parasitized brood cells. Length and diameter of nests were measured with a caliper. After eclosion, we identified all species with the help of taxonomic experts. From all nests containing a vestibular cell filled with dead ants, we randomly selected 26 for ant species identification. Voucher specimens were deposited at the University of Freiburg, Germany.

### Data analysis

Analyses were done with the software R 3.0.2 [23]. To estimate how many ant species are expected in the vestibular cells, we calculated the first-order jackknife estimator with single nests as sample units using the R-package ‘vegan’ [24]. To test if the presence of dead ants is involved in nest protection, we pooled total brood cells and parasitized brood cells per plot separately for *D. ossarium* and all other wasps combined. We used a binomial generalized linear mixed model (glmm) in the R-package ‘lme4’ [25] with a logit-link function [26]. Parasitism rate was used as response variable. Plot was treated as random factor to account for plot-specific effects on brood-cells numbers and parasitism rates. Group identity (*D. ossarium*/all other wasps) was used as explanatory variable. We also included the number of brood cells in each group as explanatory variable to account for possible effects of host population density on parasitism [27].

### Species description

The general terminology in pompilid taxonomy and morphology is based on Day [28] and its application in *Deuteragenia* (as a subgenus of *Dipogon* Fox, 1897) by Shimizu & Ishikawa [11], [16]. Specimens assigned to the *Deuteragenia conspersa*-group could not be studied, but the description and illustrations in Shimizu & Ishikawa [11], [16] are exhaustive and detailed. Thus, comparing the type series of *D. ossarium* with other members of the *D. conspersa*-group only based on published descriptions is sufficient. In addition, females and males of *D. bifasciata* (Geoffroy, 1784), as well as females of *D. subintermedia* (Magretti, 1886) and *D. vechti* (Day, 1979) have also been studied for comparison. All comparative specimens were obtained from the collection of the Museum für Naturkunde, Berlin, Germany.

### Nomenclatural Acts

The electronic edition of this article conforms to the requirements of the amended International Code of Zoological Nomenclature, and hence the new names contained herein are available under that Code from the electronic edition of this article. This published work and the nomenclatural acts it contains have been registered in ZooBank, the online registration system for the ICZN. The ZooBank LSIDs (Life Science Identifiers) can be resolved and the associated information viewed through any standard web browser by appending the LSID to the prefix “http://zoobank.org/”. The LSID for this publication is: urn:lsid:zoobank.org:pub:1ED0DD02-CF68-466C-833C-E37A3ECBAF02. The electronic edition of this work was published in a journal with an ISSN, and has been archived and is available from the following digital repositories: PubMed Central, LOCKSS.

## Results

**Figure 2:**
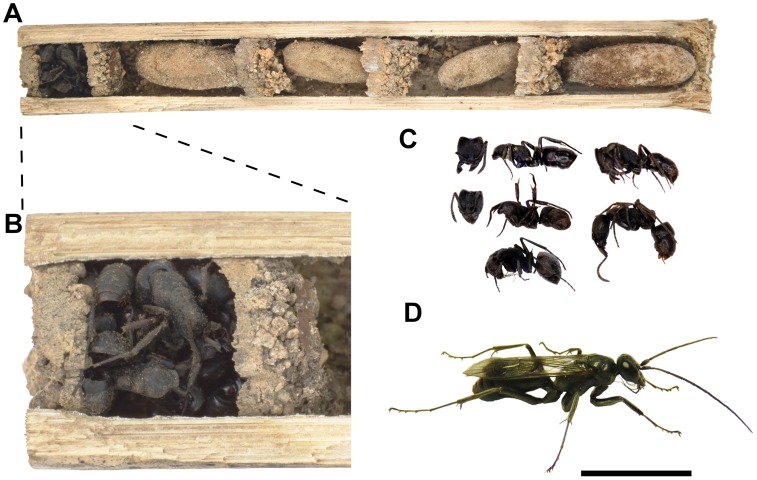
Nest protection in *Deuteragenia ossarium*. (A) Overview of a nest. Individual brood cells are separated by thin walls of soil material. (B) The nest is closed by a vestibular cell filled with dead ants. (C) Contents of a vestibular cell. *Pachycondyla astuta* was the ant species most commonly found, but other ant species, such as *Polyrhachis illaudata* Walker, 1859 (lowest ant specimen), occurred as well. (D) Freshly eclosed adult female of *D. ossarium*. Scale bar: (A) 15 mm, (B) 5 mm, (C, D) 10 mm. Photographs: Merten Ehmig (A, B), Michael Staab (C, D).

In total, we collected 829 nests of cavity-nesting wasps with 1929 brood cells belonging to 18 species (see Table 1). To our surprise, 73 of the nests (213 brood cells) had a vestibular cell filled with dead ants (Figure 2A–C). These nests contained between one and six brood cells (mean: 2.9±1.3 SD), each provisioned with a single Agelenidae spider, had a mean length of 101.2±36.8 mm, and a mean diameter of 6.6±1.6 mm.

**Table 1:** List of all sympatric cavity-nesting wasp species collected together with *Deuteragenia ossarium*.

Family	Species	Nests	Brood cells
Pompilidae	*Auplopus sp.2*	2	8
	*Auplopus sp.3*	1	4
	*Auplopus sp.4*	8	79
	*Auplopus sp.5*	2	4
	*Deuteragenia ossarium* Ohl, n. sp.	73	213
	*Deuteragenia sp.1*	4	11
	*Dipogon sp.2*	2	8
Sphecidae	*Chalybion japonicum* (Gribodo, 1883)	3	5
	*Hoplammophila aemulans* (Kohl, 1901)	156	199
Vespidae	*Allorhynchium chinense* (de Saussure, 1862)	1	1
	*Ancistrocerus nigricornis* (Curtis, 1826)	1	2
	*Ancistrocerus trifasciatus* (Müller, 1776)	4	14
	*Anterhynchium flavomarginatum* (Smith, 1852)	548	1340
	*Anterhynchium sp.1*	1	1
	*Discoelius nigriclypeus* Zhou & Li, 2013	1	1
	*Epsilon fujianensis* Lee, 1981	18	24
	*Eumenes quadratus* Smith, 1852	1	2
	*Orancistrocerus drewseni* (de Saussure, 1857)	3	13

Pompilidae were identified by Raymond Wahis (Liege, Belgium), Sphecidae by Michael Ohl (Berlin, Germany) and Vespidae by Tingjing Li (Chongqing, P.R. China).

The 26 vestibular cells examined contained up to 13 ant individuals (mean: 4.9±2.6). All ant specimens were in good condition and could be identified to nine different species (see Table S1), with up to four species co-occurring within a chamber (mean: 2.0±1.0). *Pachycondyla astuta* Smith, 1858 (Figure 2C), was the only ant in 42% of the nests, found in all but one nest, and accounted for 71% of all ant individuals. The first-order jackknife estimator predicted 13±2 ant species to occur in the vestibular cells, of which we collected 9 (69%).

**Figure 3:**
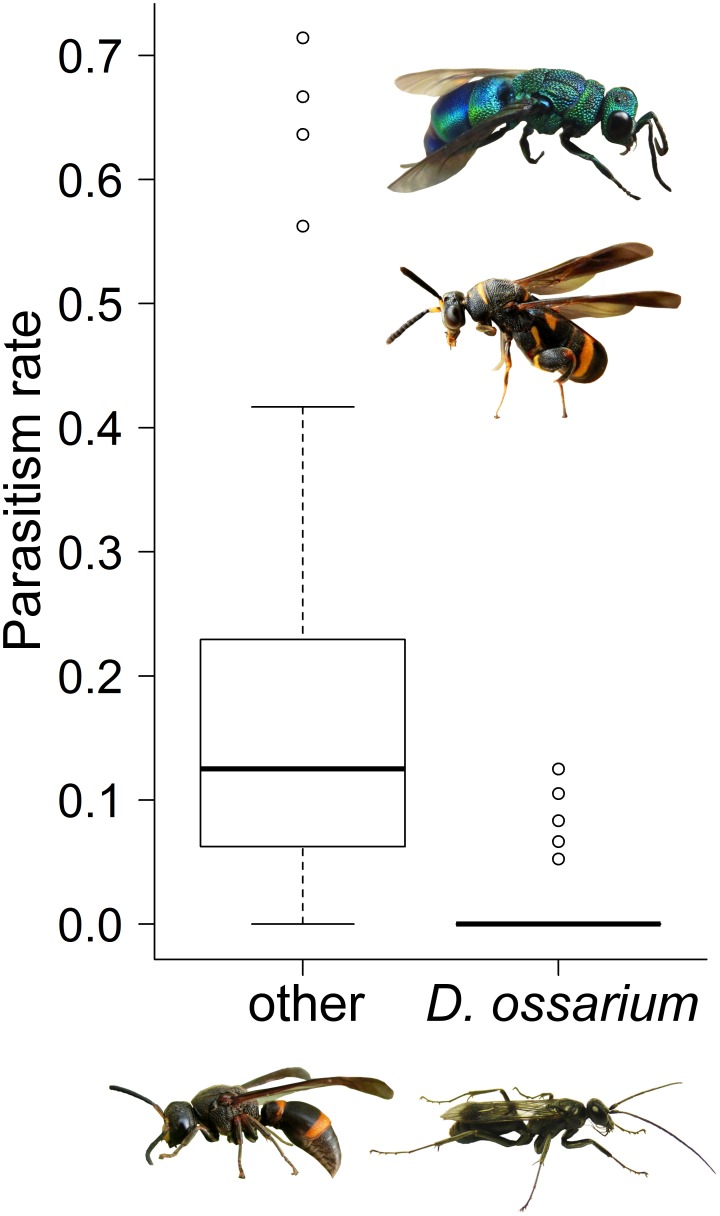
Parasitism rates of *D. ossarium* were significantly lower compared to other cavity-nesting was species. The sympatric cavity-nesting wasp community is exemplified by *Orancistrocerus drewseni* (de Saussure, 1857) (Vespidae, lower left). As examples of parasitoid species *Chrysis principalis* Smith, 1874 (Chrysididae, top) and *Leucospis sp.* (Leucospidae, middle) are shown. Photographs: Michael Staab.

Rearing of the nests with the ant-filled vestibular cell revealed an undescribed species of Pompilidae. Only seven of the 213 brood cells belonging to this species were parasitized (3%): four by an undescribed species of *Irenangulus* (Pompilidae), two by a species of minute drosophilid flies, and one by *Lycogaster violaceipennis* Chen, 1949 (Trigonalidae). The cleptoparasitic *Irenangulus sp.* and the ectoparasitoid Drosophilidae were only reared from nests of *D. ossarium*. The endoparasitoid *L. violaceipennis* was common in the nests of large-bodied Vespidae such as *Anterhynchium flavomarginatum* (Smith, 1852). Overall parasitism rates per study plot of all other trap-nesting wasp species (16.5%, Figure 3) were significantly higher than in the undescribed species and were unrelated to the number of brood cells per group (Table 2).

**Table 2:** Summary statistics of the binomial glmm testing for the effect of group identity (other wasps/*D. ossarium*) and brood cell numbers on parasitism rates.

Variable	Estimate ± SEM	z	P
Group	2.01±0.54	3.75	<0.001
Brood cells	−0.0075±0.0084	−0.89	0.37

The new species clearly belongs to the genus *Deuteragenia*, which has recently been raised from subgeneric status in *Dipogon* to full genus rank [13]. Among other diagnostics, *Deuteragenia* is characterized by the presence of a markedly long flagellomere I and an unmodified pterostigma. Within *Deuteragenia*, the new species belongs in the *Deuteragenia conspersa*-group, which has a depressed clypeal rim without preapical ridge and distinctive, long bristly setae posterolaterally on the propodeum.

### *Deuteragenia ossarium* sp. nov

**Figure 4:**
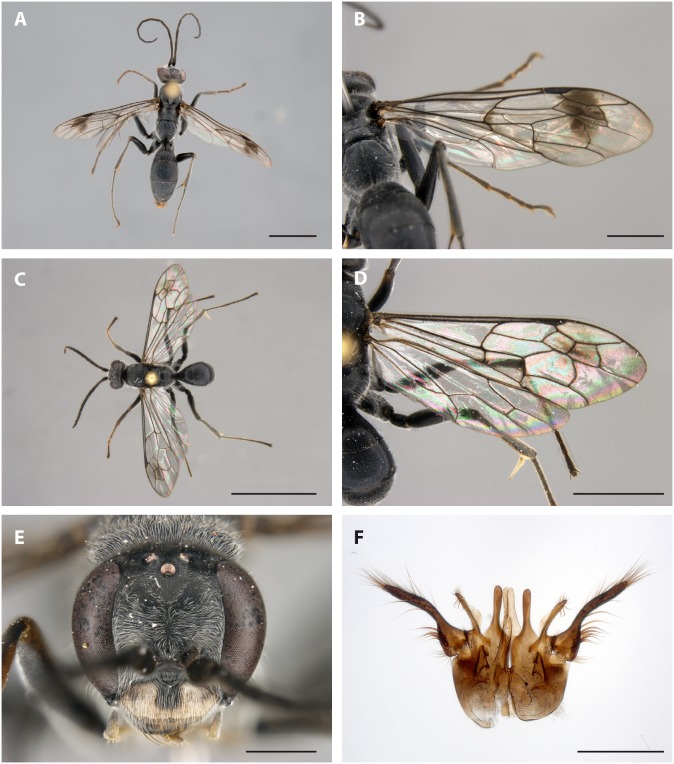
*Deuteragenia ossarium* Ohl sp. nov., (A, B) female, holotype; (C–F) male, paratype (T1482). (A, C) dorsal habitus, (B, D) right forewing, (E) head in frontal view, (F) genitalia in ventral view, slightly spread to show major elements. Scale bars: (A, C) 5.0 mm, (B, D) 2.0 mm, (E, F) 0.6 mm. Photographs: Bernhard Schurian (A–E), Birger Neuhaus (F).

Ohl, 2014 (Figure 4) urn:lsid:zoobank.org:act:9CFBF85F-04D8-4649-A1C3-2B975C993B31.

### Etymology

The new species is named after the Latin ‘ossarium’, which means bone-house or ossuary. An ‘ossarium’ is a covered site, where human remains are deposited. The species name is an allusion to the unusual nesting strategy of the new species, which closes the nest with a vestibular cell filled with dead ants. This reminds us of historical bone-houses in monasteries and graveyards, which over time were filled with piles of human bones. The new name is a noun in apposition.

### Suggested common name

As a common name for *D. ossarium* we suggest in reference to its biology the use of ‘Bone-house Wasp’.

### Distribution

Known only from South-East China.

### Diagnosis

*Deuteragenia ossarium* is most similar to *D. conspersa* (Pérez, 1905) and keys out at this species in the key to *Deuteragenia* [16] (as a subgenus in *Dipogon*). The species share the following features: Flagellomere I about 5.0x as long as wide, clypeus significantly wider than lower interocular distance, propodeal surface microsculptured and dull, mid and hind femoral venter with erect whitish setae, dorsal side of femora bare, and forewing with second recurrent vein (2 m-cu) meeting submarginal cell III at basal 0.2–0.3.

The main differences between the two species are (character states of *D. conspersa* in parentheses): body color totally pitch black (at least mandible, antenna, tarsomeres and metasomal tergum I partly ferrugineous), except for the male clypeus, which is ivory white with a black basal spot of varying size (Figure 4E) (male clypeus black, in a few specimens lateral portions pale brown), inner eye orbits rather strongly converging above: upper, middle and lower interocular distances 6.5: 10: 10 (average measures 7.2: 10: 9.3), metasomal terga II–VI with short, silvery bristles (with long brownish bristles), and propodeal surface with well-spaced, setiferous macropunctures on posterior half only (evenly finely and densely punctate, with setiferous macropunctures evenly distributed). *Deuteragenia conspersa* is known from Korea and Japan (Honshu, Shikoku, and Kyushu) [11], whereas *D. ossarium* is recorded from South-East China only.

### Description. Female

Total body length 8.9–15.2 mm, forewing length 7.4–13.3 mm.

Integument totally black. Beard and labral setae golden, ochraceous or coppery, setae otherwise mostly whitish.

Wings overall hyaline with faint greyish tinge, which is more dominant in large specimens. Forewing with narrow, indistinct fuscous marking along transverse section of at least longitudinal vein M, in some specimens also along cu-a and transverse section of Rs. Forewing also with large, fuscous marking occupying most of submarginal cells II and III, basal portion of marginal cell, and distal portion of discoidal cell II.

Frons densely, rather regularly punctate. Remaining head surface with fine, dense, shallow punctures and interspersed with widely spaced macropunctures. Pronotum and mesoscutum finely, densely punctate, mesosomal dorsum microsculptured otherwise. Mesosomal sides microsculptured, with widely spaced macropunctures. Upper part of metapleuron with transverse, coarse striae. Lateral portion of metanotum shining, with oblique striae. Propodeal dorsum miscrosculptured, with setiferous macropunctures in posterior half only. Metasoma microsculptured, dull.

Lower frons, basal half of mandible, gena, lower portion of mesosomal pleura, coxae, and posterolateral portion of propodeum with appressed, dense, silvery pubescence. Long ochraceous setae on outer mandibular surface and along anterior margin of clypeus. Gena, prosternum, forecoxa, propodeum posteriorly and tergum I with markedly long, silvery setae; similar but shorter setae on vertex, thoracic dorsum and mid and hindcoxae.

Head slightly broader than long (1.1–1.3). Inner eye orbits rather strongly converging above: upper, middle and lower interocular distances 6.5: 10: 10. Length of flagellomere I 4.9–5.4x as long as wide.

Structurally otherwise apparently identical to *D. conspersa* (see Shimizu & Ishikawa [11]).

### Male

Overall similar to female, except for: Total body length 6.6–9.8 mm, forewing length 5.9–8.3 mm.

Integument totally black, except for the following: clypeus ivory white with black basal marking of varying size; labial and maxillary palps, tibial spurs and foretibia and tarsi below ochraceous. In some specimens, pronotum with brownish transverse band. Body setae mostly whitish.

Wings hyaline, markings absent except for faint longitudinal marking in submarginal cells II and III, in a few larger males also in marginal cell and discoidal cell I.

Genitalia (Figure 4F): Parameres with long, thin setae in apical two-thirds and strong, stout setae ventrobasally. Parapenial lobes slightly extending beyond apex of aedeagus, finger-shaped. Aedeagus weakly sclerotized, simple. Digitus laterally compressed, apex strongly setose. Cuspis indistinct.

Structurally otherwise apparently identical to *D. conspersa* (see Shimizu & Ishikawa [11]).

### Material examined

**Holotype**, female. **CHINA**, **Zhejiang Province**, ca. 30 km NW of Kaihua, 29° 16′ 50″N/118° 5′ 2″E, 655 m, 5 Jun 2012, leg. M. Staab (T487) (Insect Collection of the Institute of Zoology, Chinese Academy of Sciences, Beijing, China).

**Paratypes** (61 females, 37 males). **CHINA. Zhejiang Province**, ca. 30 km NW of Kaihua, 29° 14′ 47″N/118° 6′ 58″E, 402 m, 2 Jun 2012, leg. M. Staab (T482, T495, T514) (1 female, 2 males); same data, but 29° 16′ 53″N/118° 5′ 17″E, 679 m, 05 Jun 2012 (T538, T539) (3 females, 1 male); same data, but 29° 16′ 14″N/118° 4′ 51″E, 566 m, 05 Jun 2012 (T542) (2 females, 1 male); same data, but 29° 16′ 53″N/118° 5′ 17″E, 679 m, 29 Jun 2012 (T532) (1 female); same data, but 29° 16′ 53″N/118° 5′ 17″E, 679 m, 3 Jul 2012 (T529, T530) (1 female, 1 male); same data, but 29° 16′ 50″N/118° 5′ 2″E, 655 m, 30 Sep 2012 (T724) (2 females); same data, but 29° 14′ 49″N/118°6′ 44″E, 507 m, 04 Oct 2012 (T896) (1 female); same data, but 29° 16′ 53″N/118° 5′ 17″E, 679 m, 06 Oct 2012 (T771, T772) (2 females); same data, but 29° 16′ 53″N/118° 5′ 17″E, 679 m, 07 Oct 2012 (T770) (1 male); same data, but 29° 15′ 7″N/118° 8′ 37″E, 903 m, 8 Oct 2012 (T1546) (4 females, 2 males); same data, but 29° 14′ 20″N 7 118°7′ 26″E, 720 m, 9 Oct 2012 (T1288, T1289, T1290, T1291, T1292) (3 females, 3 males); same data, but 29° 16′ 37″N/118° 5′ 26″E, 617 m, 10 Oct 2012 (T1273, T1278, T1279, T1281) (5 females, 1 male); same data, but 29° 16′ 50″N/118° 5′ 2″E, 655 m, 17 Oct 2012 (T1453) (2 females); same data, but 29° 15′ 18″N/118° 8′ 51″E, 880 m, 21 Oct 2012 (T1835) (1 female); same data, but 29° 16′ 50″N/118° 5′ 2″E, 655 m, 9 May 2013 (T1374) (1 male); same data, but 29° 12′ 54″N/118°7′ 18″E, 251 m, 12 May 2013 (T1664) (1 female); same data, but 29° 14′ 57″N/118°8′ 5″E, 590 m, 12 May 2013 (T1820) (1 female, 1 male); same data, but 29° 14′ 58″N/118° 8′ 7″E, 639 m, 12 May 2013 (T1855) (1 male); same data, but 29° 16′ 37″N/118° 5′ 26″E, 617 m, 13 May 2013 (T0729) (1 female, 1 male); same data, but 29° 16′ 14″N/118° 4′ 51″E, 566 m, 13 May 2013 (T1301) (1 male); same data, but 29° 15′ 7″N/118° 8′ 37″E, 903 m, 13 May 2013 (T1371) (2 females, 1 male); same data, but 29° 16′ 50″N/118° 5′ 2″E, 655 m, 13 May 2013 (T1375, T1470) (1 female, 1 male); same data, but 29° 14′ 20″N 7 118°7′ 26″E, 720 m, 13 May 2013 (T1445, T1565, T1601) (1 female, 3 male); same data, but 29° 15′ 7″N/118° 8′ 37″E, 903 m, 13 May 2013 (T1481, T1482) (1 female, 2 male); same data, but 29° 15′ 18″N/118° 8′ 51″E, 880 m, 13 May 2013 (T1499) (1 female, 2 males); same data, but 29° 12′ 54″N/118°7′ 18″E, 251 m, 13 May 2013 (T1511, T1528) (2 females); same data, but 29° 15′ 7″N/118°9′ 28″E, 670 m, 13 May 2013 (T1539, T1833) (1 female, 3 males); same data, but 29° 12′ 52″N/118°8′ 14″E, 419 m, 13 May 2013 (T1572) (1 female, 1 male); same data, but 29° 14′ 57″N/118°8′ 5″E, 590 m, 13 May 2013 (T1821, T1914, T1915, T1916, T1917, T1918) (7 females, 1 male); same data, but 29° 14′ 58″N/118° 8′ 7″E, 639 m, 13 May 2013 (T1853) (1 female); same data, but 29° 14′ 47″N/118° 6′ 58″E, 402 m, 13 May 2013 (T1920) (2 female, 2 male); same data, but 29° 14′ 20″N 7 118°7′ 26″E, 720 m, 15 May 2013 (T1520) (1 female); same data, but 29° 14′ 20″N 7 118°7′ 26″E, 720 m, 16 May 2013 (T0684) (1 female); same data, but 29° 16′ 14″N/118° 4′ 51″E, 566 m, 16 May 2013 (T1302) (1 female, 1 male); same data, but 29° 12′ 54″N/118°7′ 18″E, 251 m, 16 May 2013 (T1508) (1 female); same data, but 29° 15′ 7″N/118°9′ 28″E, 670 m, 16 May 2013 (T1537) (1 male); same data, but 29° 15′ 7″N/118°9′ 28″E, 670 m, 17 May 2013 (T1540) (1 female); same data, but 29° 14′ 20″N 7 118°7′ 26″E, 720 m, 17 May 2013 (T1566) (1 female). **Jiangxi Province**, ca. 15 km SE of Wuyuan, 29° 7′ 16″N/117° 54′ 22″E, 125 m, 16 May 2013 (T2402) (1 female, 1 male); same data, but 29° 7′ 30″N/117° 54′ 31″E, 221 m, 17 May 2013 (T2197) (1 female); same data, but 29° 7′ 31″N/117° 54′ 36″E, 247 m, 2 Jun 2013 (T2540) (1 female, 1 male); same data, but 29° 7′ 31″N/117° 54′ 36″E, 247 m, 3 Jun 2013 (T2541) (1 male).

All specimens have been reared from trap nests (Figure 1).

The majority of the paratypes will be deposited in the Insect Collection of the Institute of Zoology, Chinese Academy of Sciences, Beijing, China, along with the holotype. The remaining paratypes will finally remain in the collections of the Museum für Naturkunde, Berlin, Germany, and pairs will also be deposited in the Natural History Museum, London, UK, the American Museum of Natural History, New York, USA, the Smithsonian Institution, Washington DC, USA, the California Academy of Sciences, San Francisco, USA, the Utah State University, Logan, USA, and the collection of the Institute of Earth and Environmental Sciences, University of Freiburg, Germany.

## Discussion

We report here on a unique and effective nest-protecting strategy, the construction of a vestibular cell filled with dead ants in a new spider wasp. Vestibular cells occur commonly in solitary Hymenoptera [10], are normally empty but their function is not known. At least two studies that each focus on a single species in a single locality indicate that vestibular cells may not serve a protective function [29], [30]. However, offspring of *D. ossarium* had significantly lower parasitism rates than the sympatric cavity-nesting wasp community. Low parasitism was unrelated to population density effects [27], thus delivering initial support for the protective function of the ant-filled vestibular cell. We propose two non-exclusive hypotheses: chemical camouflage and chemical defense by utilization of volatile chemical cues emanating from the dead ants. Ants produce a diverse array of organic compounds [31], including species-specific cuticular hydrocarbons (CCHs) which are a central part of the nestmate-recognition system [32]–[34]. Being long-chained molecules of low volatility, CCHs persist on the cuticula of dead Hymenoptera for a long time period [35], thus giving *D. ossarium* nests the scent of an ant colony.

This scent camouflages nests against natural enemies which search their host by scent, as CCHs are known to release behavioral reactions without tactile interactions [32]. The scent may also repel predators, as most ant species ferociously defend their colonies against intruders. In this context, we find it particularly interesting that the numerically dominating ant species in *D. ossarium* nests is *P. astuta*, an aggressive, large-bodied, and common species in the study region that has a powerful sting. By using a big ant species, already few ant individuals are sufficient to stock the vestibular cell with large quantities of CCHs. By using an abundant ant species, potential predators may have had contact with the species before and therefore avoid the species-specific scent. The proposed function of the ant-chamber is most effective against predators that break the nest and against parasitic wasps that penetrate the nest with their long ovipositor. Such parasitoids attacked other trap-nesting wasps including other Pompilidae in the study region and may also attack *D. ossarium* by penetrating the wood that contains naturally the nest cavity. However, such species were never found in nests of *D. ossarium*, which was only attacked by parasitoids which entered the nest prior to the construction of the ant chamber.

Although some cavity-nesting wasp species are known to incorporate arthropod fragments into nest construction [36] or nest camouflage [37], and to prey on ants [38], *D. ossarium* is the first species that uses complete ants for nest construction but by far not the only animal species that exploits ants to protect their progeny. Many other species independently evolved stunning strategies; for example the caterpillars of most Lycaenidae butterflies life obligatory in ant colonies [39], many beetles are exclusively known from the safety of ant nests [40] and several snakes lay their eggs only in the fungus-chambers of aggressive leaf-cutting ant nests [41]. However, none of these examples builds literally a barrier out of ant carcasses. When we first saw a nest of *D. ossarium* we were reminded on the historical bone houses or ossuaries in graveyards, and thus suggest in allusion to its biology ‘Bone-house Wasp’ as common name.

## Supporting Information

Table S1 **Ant species found in 26 vestibular cells of** ***Deuteragenia ossarium*** **.** Species were identified by Michael Staab (Freiburg, Germany).
(DOCX)
