# Supplementary material for: A Unique Nest-Protection Strategy in a New Species of Spider Wasp
Source: PLoS One. 2014 Jul 2;9(7):e101592. doi: 10.1371/journal.pone.0101592 (PMC4079592; doi:10.1371/journal.pone.0101592)
Supplement: Table S1 — Ant species found in 26 vestibular cells of Deuteragenia ossarium . Species were identified by Michael Staab (Freiburg, Germany). (DOCX) [file pone.0101592.s001.docx]

**Table S1.** Ant species found in 26 vestibular cells of *Deuteragenia ossarium*. Species were identified by Michael Staab (Freiburg, Germany).

| Subfamily | Species | Abundance | Occurrence |
| --- | --- | --- | --- |
| Dolichoderinae | *Technomyrmex obscurior* Wheeler, 1928 | 1 | 1 |
| Ectatomminae | *Gnamptogenys panda* (Brown, 1948) | 10 | 7 |
| Formicinae | *Camponotus pseudoirritans* Wang, Xiao & Wu, 1989 | 1 | 1 |
|  | *Camponotus rubidus* Wang, Xiao & Wu, 1989 | 1 | 1 |
|  | *Polyrhachis illaudata* Walker, 1859 | 10 | 7 |
|  | *Polyrhachis lamellidens* Smith, 1874 | 1 | 1 |
| Ponerinae | *Leptogenys kitteli* (Mayr, 1870) | 2 | 2 |
|  | *Pachycondyla astuta* Smith, 1858 | 90 | 25 |
|  | *Pachycondyla chinensis* (Emery, 1895) | 10 | 7 |
